# MC-LR Exposure Leads to Subfertility of Female Mice and Induces Oxidative Stress in Granulosa Cells

**DOI:** 10.3390/toxins7124872

**Published:** 2015-12-02

**Authors:** Jiang Wu, Mingming Yuan, Yuefeng Song, Feng Sun, Xiaodong Han

**Affiliations:** 1 Immunology and Reproduction Biology Laboratory and State Key Laboratory of Analytical Chemistry for Life Science, Medical School, Nanjing University, Nanjing 210093, Jiangsu, China; wujiang@nju.edu.cn (J.W.); ymmslim@outlook.com (M.Y.); b101230061@smail.nju.edu.cn (Y.S.); medsunfeng@smail.nju.edu.cn (F.S.); 2 Jiangsu Key Laboratory of Molecular Medicine, Nanjing University, Nanjing 210093, Jiangsu, China

**Keywords:** MC-LR, follicle atresia, female subfertility, granulosa cells, oxidative stress

## Abstract

Health risk of human exposure to microcystin-leucine arginine (MC-LR) has aroused more and more attention over the past few decades. In the present study, MC-LR was orally administered to female mice at 0, 1, 10 and 40 μg/L for three and six months. We found that chronic exposure to MC-LR at environmental levels could stimulate follicle atresia and lead to decreased developmental follicles, accompanied by a reduction of gonadosomatic index (GSI). In line with the irregular gonadal hormone level and estrus cycles, subfertility of female mice was also confirmed by analyzing numbers of litters and pups. The *in vitro* study suggested that granulosa cells could uptake MC-LR and should be the target of the toxicant. Oxidative stress in granulose cells induced by MC-LR promoted follicle atresia and eventually leads to female subfertility.

## 1. Introduction

In the past few decades, eutrophication of water ecosystems caused by growing farming practices, detergent usage, and sewage generation resulted in a sustained proliferation of cyanobacteria [1]. The frequency and intensity of cyanobacteria blooms is reported to increase throughout the world [2]. These blooms can release toxic metabolites and directly affect the quality of drinking water [3]. In respect to toxin production, microcystins (MCs) are acknowledged as the most ubiquitous cyanotoxins. MCs are cyclic heptapeptides composed of over 100 congeners, differing mainly in their conformation, methylation and peptide sequence of the molecule [4,5]. Among these varied congeners, MC-LR receives widespread attention due to its abundance and strong toxicity. Studies reported that MC-LR accounts for 46%–99.8% of the total MCs [6,7] and varies from 0.14 to 13,000 μg/L in surface waters of United States [8,9,10].

MC-LR is highly water-stable and resistant to boiling, chemical hydrolysis or oxidation at near-neutral pH and conventional water treatment processes such as flocculation, sedimentation, sand filtration, and chlorination and treatment processes, which include potassium permanganate or chlorine, cannot remove all of the toxicant [11,12,13]. MC-LR presents potential health threats for humans mainly by oral ingestion, especially through drinking water [14,15]. Not surprisingly, MC-LR has been detected in chronically-exposed human population’s serum and the concentration reached as high as 0.39 μg/L [16]. These health threats have already led the World Health Organization (WHO) to set a provisional guideline value for MC-LR of 1 μg/L in drinking water [17]. However, recent data have revealed that the concentration could exceeding this recommendation in the raw and treated drinking water samples collected from water treatment plants [18,19].

MC-LR has been well documented to arouse hepatotoxicity, neurotoxicity, dermatoxicity, and gastrointestinal disorders [20,21,22]. By contrast, reports about female reproductive toxicity of MC-LR are limited, especially on mammals. In our previous acute toxicity experiment, pathomorphological changes of ovary and disturbance of estrus cycle in mice were observed after four-week administration of 20 μg/kg MC-LR by intraperitoneal (i.p.) injection. Moreover, MC-LR was detected in the ovary tissue as expected [23]. For humans, however, chronic exposure to low-dose MC-LR in drinking water is the major risk of this toxicant. Therefore, in our present study, MC-LR was delivered to mice in drinking water for three or six months. The doses chosen were 0, 1, 10, and 40 μg/L according to the WHO guideline and environmental levels. Female reproductive toxicity of MC-LR was evaluated after the treatment.

As the basic functional unit of the ovary, each follicle is composed of an oocyte surrounded by one or more layers of somatic granulosa cells. Granulosa cells are responsible for secretion of steroid hormones which are necessary to prepare the reproductive tract for fertilization and the establishment of pregnancy [24]. Thus, in order to explore possible action mechanism of MC-LR, primary cultured mouse granulosa cells (mGCs) were used in the present *in vitro* study.

## 2. Results

### 2.1. Reduction in GSI

**Figure 1:**
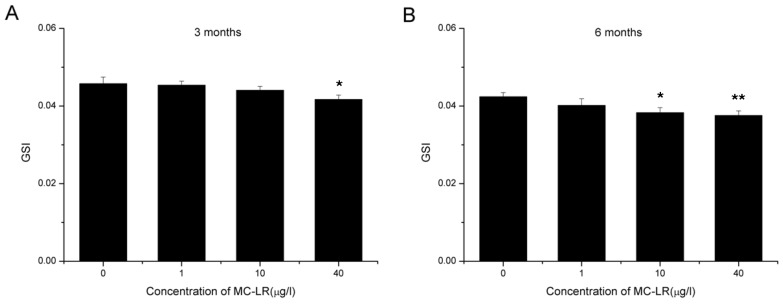
GSI in female mice exposed orally to 0, 1, 10 and 40 μg/L MC-LR for three (**A**) or six months (**B**). Data are shown as mean ± S.E. Asterisk denotes a response that is significantly different from the control. *n* = 10, * *p* < 0.05; ** *p* < 0.01.

In the three months MC-LR exposure cohort, ovaries collected from control group mice had an average GSI of 0.0458 ± 0.0017, while which in treated mice with 1, 10, and 40 μg/L MC-LR were 0.0454 ± 0.0010, 0.0441 ± 0.0010, and 0.0417 ± 0.0011, respectively. The difference between 40 μg/L MC-LR treated groups and control group were statistically significant (Figure 1A). In the six months MC-LR exposure cohort, ovaries of control group mice had an average GSI of 0.0424 ± 0.0011, while which in treated mice with 1, 10 and 40 μg/L MC-LR were 0.0402 ± 0.0017, 0.0383 ± 0.0013, and 0.0376 ± 0.0012, respectively. Compared with control group, GSI in 10 and 40 μg/L MC-LR groups significantly decreased (Figure 1B). This loss in GSI was dose and time dependent, and could be ascribed to pathomorphological changes in treated ovaries induced by MC-LR.

### 2.2. Accelerated Follicle Atresia and Loss of Developing Follicles

**Figure 2:**
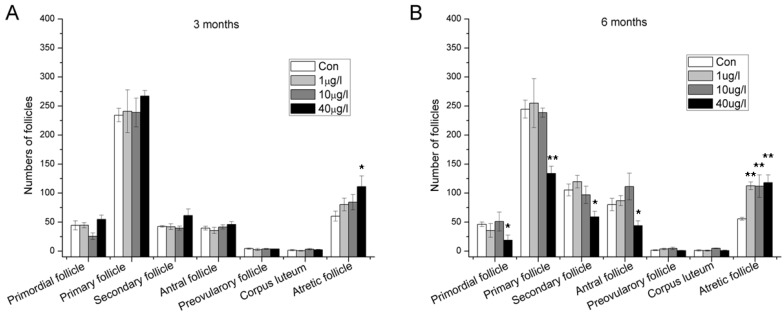
Number of different types of follicles in the ovaries of female mice exposed orally to 0, 1, 10 and 40 μg/L MC-LR for three (**A**) or six months (**B**). (mean ± S.E., *n* = 10, * *p* < 0.05; ** *p* < 0.01).

The effects of different concentrations of MC-LR on the ovaries determined by histopathological observations are shown in Figure 2. After three months MC-LR exposure by drinking water, atretic follicles increased evidently at a high dose (40 μg/L) of MC-LR while no obvious changes were observed in other categories of follicles (Figure 2A). After six months MC-LR exposure, accelerated follicle atresia was also observed at all treated group. Meanwhile, primordial, primary, secondary, and antral follicles decreased significantly in high dose group (Figure 2B). Loss of these developing follicles probably resulted from continuous accelerated follicle atresia.

### 2.3. Decline of Serum Estradiol and Ascending of Serum Progesterone

**Figure 3:**
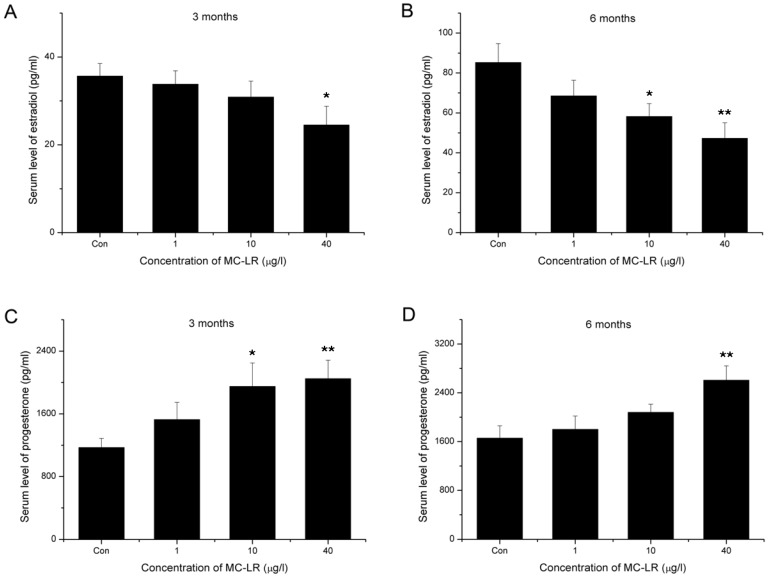
The serum level of estradiol (**A**,**B**) and progesterone (**C**,**D**) after exposure orally to 0, 1, 10 and 40 μg/L MC-LR for three or six months. (mean ± S.E., *n* = 10, * *p* < 0.05; ** *p* < 0.01).

The changes of steroid hormones in the serum were shown in Figure 3. The results indicate that after exposure to 40 μg/L MC-LR for three months, serum estradiol significantly decreased (Figure 3A). After six months, serum estradiol significantly decreased in 10 and 40 μg/L MC-LR treated groups (Figure 3B). Serum progesterone, on the other hand, increased significantly in the high dose MC-LR treated groups, both in three and six months cohorts (Figure 3C,D).

### 2.4. Disorder of Estrous Cycle

**Figure 4:**
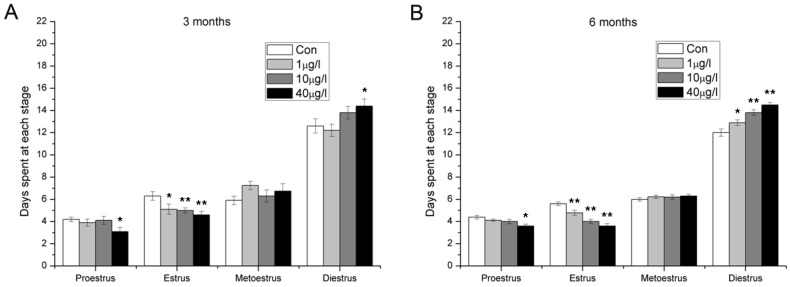
The stages of estrus cycle after exposure orally to 0, 1, 10 and 40 μg/L MC-LR for three (**A**) or six months (**B**). (mean ± S.E., *n* = 10, * *p* < 0.05; ** *p* < 0.01).

Abnormal estrous cycles were observed after exposure to MC-LR (Figure 4). Both in three months and six months exposure cohorts, estrus was shortened in all the MC-LR treated group and the duration of proestrus stage decreased in the high dose group. In addition, extended diestrus were observed after six months MC-LR exposure.

### 2.5. Subfertility Induced by MC-LR

When MC-LR treated female mice were mated to non-treated males, we observed MC-LR treated females to be less fertile relative to their control counterparts (Table 1 and Table 2). This subfertility included a growth in stillbirth rate and a reduction in number of living pups per litter. Specifically, significant growth of stillbirth rate was observed in both 10 and 40 μg/L MC-LR treated group. The reduction in number of living pups per litter showed dose and time dependent. After three months treatment, significant reduction was observed in 40 μg/L MC-LR treated group and the reduction in 1 and 10 μg/L groups was not significant. After six months administration, significant reduction was observed in both 10 and 40 μg/L groups.

**Table 1:** Effects of three months exposure to 0, 1, 10 and 40 μg/L MC-LR on female mice fertility.

MC-LR (μg/L)	N	Litter	Living Pups	Dead Pups	Stillbirth Rate	Living Pups/Litter
0	10	40	261	4	1.32 ± 0.62	6.53 ± 0.40
1	10	40	220	6	2.19 ± 0.95	5.50 ± 0.40
10	10	38	228	15	5.71 ± 1.75 *	6.00 ± 0.48
40	10	34	171	15	8.63 ± 2.12 **	5.03 ± 0.64 *

Stillbirth rate = (dead pups/total pups) × 100% in each litter. Values are mean ± S.E. *versus* controls. *n* = 10, * *p* < 0.05; ** *p* < 0.01.

**Table 2:** Effects of six months exposure to 0, 1, 10 and 40 μg/L MC-LR on female mice fertility.

MC-LR (μg/L)	N	Litter	Living Pups	Dead Pups	Stillbirth Rate	Living Pups/Litter
0	10	36	168	4	1.75 ± 1.09	4.67 ± 0.51
1	10	28	117	8	4.59 ± 2.05	4.18 ± 0.54
10	10	34	112	14	10.20 ± 3.64 *	3.29 ± 0.49 *
40	10	32	106	15	9.18 ± 2.36 *	3.31 ± 0.38 *

Stillbirth rate = (dead pups/total pups) × 100% in each litter. Values are mean ± S.E. *versus* controls. *n* = 10, * *p* < 0.05; ** *p* < 0.01.

### 2.6. MC-LR Affects Cell Viability of mGCs

**Figure 5:**
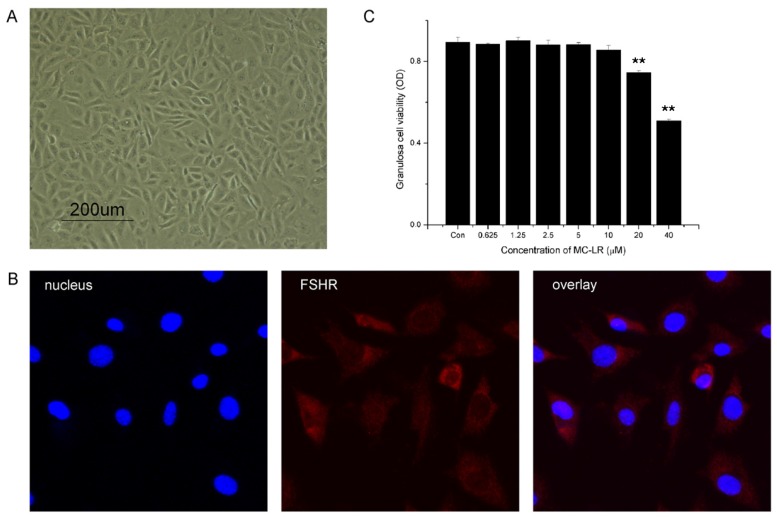
MC-LR affects cell viability of mGCs. (**A**) Primary mGCs were obtained from mouse ovary and an optical microscope image was taken; (**B**) mGCs cultured on coverslips were fixed and immunostained with specific antibodies for FSHR; (**C**) effects of 48 h exposure to MC-LR on mGCs cell viability. mGCs were treated with 0, 0.625, 1.25, 2.5, 5, 10, 20 and 40 μM MC-LR, respectively. 48 h later, measurement of cell viability was carried out with CCK-8. The percentage of dead cells was increased in a dose-dependent manner. The analysis were performed with three replications and representative data was shown. (** *p* < 0.01).

The identity of mGCs was defined by morphology and FSHR expression (Figure 5A,B). The results showed that cell viability was significantly decreased after exposure to MC-LR for 48 h at a concentration of 20 μM or higher. No significant differences were observed in groups exposed to 10 μM or lower dose.

### 2.7. MC-LR can Enter mGCs

**Figure 6:**
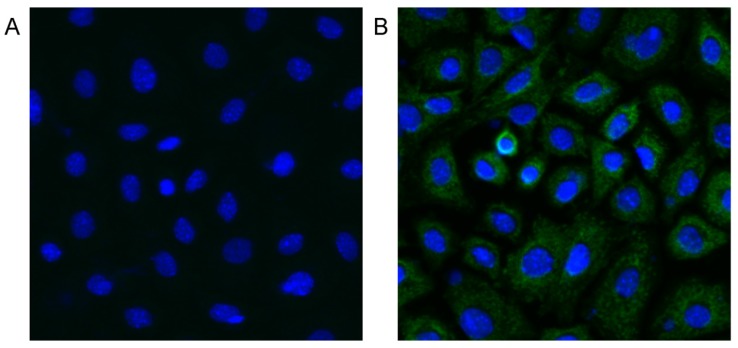
Immunofluorescence detection for MC-LR in primary cultured mGCs after 48 h exposure to 0 (**A**) and 1.25 μM MC-LR (**B**). Green fluorescence represents MC-LR positive, and blue fluorescence represents nuclei (DAPI staining positive). After 48 h exposure to 1.25 μM MC-LR, MC-LR can be observed in the cytoplasm of mGCs.

After 48 h exposure to 1.25 μM MC-LR, immunofluorescence assay was performed to determine whether MC-LR could get into mGCs. Green fluorescence was detected in MC-LR treated mGCs but not in control mGCs (Figure 6), and this demonstrated that MC-LR is able to enter mGCs.

### 2.8. Increased Lipid Peroxidation and Inhibition of Antioxidant Enzymes Activity

**Figure 7:**
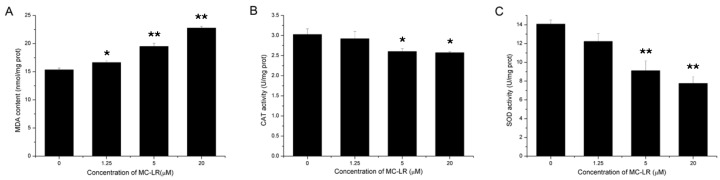
Effects of 48 h exposure to MC-LR on mGCs lipid peroxidation (**A**) and antioxidant enzymes activity (**B**,**C**). The analysis were performed with three replications and representative data was shown. (* *p* < 0.05; ** *p* < 0.01).

After 48 h MC-LR exposure, lipid peroxidation significantly increased which were supported by evaluated malondialdehyde (MDA) content in all the treatment groups. Meanwhile, catalase (CAT) and superoxide dismutase (SOD) activity were significantly reduced after the treatment of 5 and 20 μM MC-LR (Figure 7). These results suggested that MC-LR could induce lipid peroxidation in mGCs, and oxidative stress occurred in MC-LR challenged mGCs.

## 3. Discussion

There are increasing concerns about the health risk of human exposure to MC-LR over the past decades. Nevertheless, most studies focused on the hepatotoxicity, neurotoxicity, or kidney impairment induced by MC-LR, and reports about its female reproductive toxicity are quite limited.

In the present study, after three months administration of MC-LR through drinking water, the GSI of female Balb/c mice significantly decreased in the 40 μg/L group. When prolonging the treatment to six months, the reduction of GSI became more dramatic. Generally speaking, the reduction of ovary weight goes together with the pathomorphological changes. As ovarian follicles are the basic functional unit and account for the main part of ovary, we subsequently performed the histological evaluation of follicles. We showed that continuous exposure to MC-LR led to promoted follicle atresia and decreased primordial, primary, secondary, and antral follicles. Follicle atresia is a natural physiological process which occurs throughout female reproductive life during all stages of follicular development. The delicate balance between follicular atresia and follicle growth is central for normal ovarian function [25,26,27]. Thus, destruction of this balance induced by MC-LR indicated dysfunction of ovary.

As one of the key ovarian functions is to secret estradiol and progesterone, we used ELISA kits to detect serum steroid hormones. The data showed that continuous exposure to environmental level MC-LR resulted in decreased estradiol and increased progesterone. Inconsistent with this, serum progesterone decreased after four-week administration of 5 and 20 μg/kg MC-LR by i.p. injection in our previous acute toxicity study. These differences may be attributable to between-study differences in dose, periods, and route of administration of MC-LR. In the present study, chronic oral exposure of natural concentration levels of MC-LR was seemed to disturb steroidogenesis of ovarian granulosa cells, which are responsible for production of estradiol and progesterone. Recent studies demonstrated that mitogen-activated protein kinases (MAPKs) activation is involved in the regulation of steroidogenesis in granulosa cells [28,29]. Meanwhile, Masaharu *et al.* reported that MC-LR could activate MAPKs in HEK293 cells [30]. Further studies are needed to investigate whether MAPKs activation also plays an important part in MC-LR-induced steroidogenesis disorder in granulosa cells. Estradiol and progesterone play pivotal roles during the follicular and luteal phases of the menstrual cycle [31]. Connected to changes of serum hormones, disorder of estrus cycles was also observed. Importantly, MC-LR exposure led to shortened estrus and prolonged distrus. In the midday of proestrus, the rising level of estradiol peaks and acts on the hypothalamus and pituitary, triggering the ovulatory surge of luteinizing hormone. The surge facilitates ovarian follicular maturation and precedes the ovulatory rupture of the follicle [32]. As the female is sexually receptive through late proestrus to estrus [33,34,35], shortened estrus and prolonged diestrus would naturally lead to decreasing mating frequency and decline in fertility. This speculation was confirmed by fertility assessment, which showed that MC-LR exposure resulted in a growth in stillbirth rate and a reduction in number of living pups per litter.

Being the main resource of estradiol and progesterone, granulosa cells could regulate ovarian follicle development by secreting various factors, including gonadal steriods, growth factors, and cytokines. Moreover, granulosa cells were demonstrated to play a role as the initiator of follicle atresia [34]. The accelarated follicle atresia, disorder of estrus cycles, and reproductive subfertility induced by MC-LR suggested granulosa cells were quite likely to be the target of MC-LR. Therefore, we performed *in vitro* study to investigate the influences of MC-LR on mGCs.

After 48 h exposure to 20 or 40 μM MC-LR, cell viability of mGCs significantly decreased. However, MC-LR is unable to easily penetrate biological membranes on account of its structure and molecular weight (995 Da) [36]. In fact, some kinds of cells could express specific membrane transporters and enable MC-LR accumulation. Such transporters handling intracellular transport of cyclic peptides are called organic anion transporting polypeptides (Oatps) [37,38]. In the present study, after 48 h exposure, MC-LR was detected in mGCs by immunofluorescence assay. This suggested Oatps are also expressed in mGCs. Expression of Oatp isoforms varies markedly across tissue types. It has already established that Oatp1a4, Oatp1a5, Oatp2a1, Oatp2b1, Oatp3a1, and Oatp5a1 express in the mouse ovary [39]. However, which kinds of Oatps are present in mGCs still remains unclear. Research has demonstrated that oxidative stress plays an important role in MC-LR toxicity [40,41]. MC-LR could induce oxidative stress through generation of reactive oxygen species (ROS) and oxidative damage products such as lipid peroxides [42]. In the current study, evaluated MDA content suggested lipid peroxidation did increase after 24 h MC-LR exposure. Consistent with this, activity of antioxidant enzymes such as CAT and SOD significantly decreased. These results confirmed that oxidative stress may be a crucial mechanism of MC-LR cytotoxicity in mGCs. However, further work is required to explore the underlying mechanisms of this process. One of the most studied pathways of MC-LR activity is inhibition of protein phosphatase (PP) 1 and PP2A. Transported by Oatps, MC-LR could interact with the catalytic subunits of PP1 or PP2A and subsequently induces oxidative stress [43].

## 4. Experimental Section

### 4.1. Chemicals and Reagents

MC-LR (ALX-350-012-M001, isolated from *Microcystis aeruginosa*, purity ≥95%, identity determined by MS) was purchased from Alexis Biochemicals (Lausen, Switzerland). MC-LR (1 mg) was dissolved in 100 μL DMSO and diluted to 1 mL with sterilized water to prepare the stock solution (1 g/L). Stock solution was stable for up to six months according to the work sheet of the supplier. Dulbecco’s modified Eagle’s medium-Ham’s F-12 (DMEM-F12) medium, bovine serum albumin (BSA), trypsin, and 4,6-diamidino-2-phenylindole (DAPI) were purchased from Sigma-Aldrich Inc. (St. Louis, MO, USA). Mouse monoclonal antibody to microcystin-LR (MC10E7), estradiol and progesterone ELISA kit were purchased from Enzo Life Sciences (Lausen, Switzerland). Goat polyclonal antibody to Follicle-stimulating Hormone Receptor (FSHR) was purchased from Santa Cruz Biotechnology Inc. (Santa Cruz, CA, USA). Alexa Fluor^®^ 594 donkey anti-mouse IgG (H+L) was obtained from Invitrogen Co. (Grand Island, NY, USA). Cell Counting Kit-8 (CCK-8) was obtained from Dojindo Molecular Technologies, Inc. (Kumamoto, Japan). MDA, CAT and SOD assay kits were from Beyotime Biotechnology (Nanjing, China).

### 4.2. Animals and Treatment

For *in vivo* study, 4-week old specific pathogen free (SPF) female BALB/c mice (*n* = 240) and proven breeder male BALB/c mice were purchased from Experimental Animal Center of the Academy of Military Medical Science Institute, China. Animals were housed in the animal facility of School of Medicine, Nanjing University with a 12 h: 12 h light-dark cycle. Animals were handled following the directions in the ethics approval obtained from the Experimentation Ethics Review Committee of Nanjing University (ethics approval no. A9089). Animals were kept and accommodated for one week before experiments.

Mice were randomly divided into two cohorts (3 months and 6 months). Each cohort had four groups: one control and three experimental groups with 30 mice in each group. In the experimental groups, MC-LR stock solution was diluted to 1, 10, and 40 μg/L with sterilized water to prepare the drinking water used for exposure. The drinking water was prepared every week and mice were exposed orally at 1, 10, and 40 μg/L MC-LR for consecutive three or six months. The control animals received the sterilized water with 0.0004% DMSO. Dose selection was based on the recommended level of MCs (1 μg/L) by WHO for drinking water, and the highest dose of MC-LR at 40 μg/L which can often be reached in natural water body [44]. Of each group, 20 mice were kept alive for estrous cycle monitoring (*n* = 10) or fertility assessment (*n* = 10), the other 10 mice were executed for gonadosomatic index (GSI) calculating, serum hormone assay, and histological evaluation of follicles.

### 4.3. GSI Calculation

Mice were weighed before and after experiment. After the treatment, the bilateral ovaries of the female mice were excised and weighed. GSI was calculated by the formula: GSI = weight of ovaries/body weight × 100.

### 4.4. Histological Evaluation of Follicles

Following the administration of MC-LR, ovaries were placed in Bouin’s fixative for 4 h, washed in 70% ethanol, paraffin embedded, and serially sectioned (5 μm thick) throughout the entire ovary, with every fifth slide counterstained with hematoxylin and eosin. Classification of mouse follicles was according to Myers M’s classification scheme [45]. Briefly, follicles were classified as primordial if they contained an oocyte surrounded by a partial or complete layer of squamous granulosa cells. Primary follicles showed a single layer of cuboidal granulosa cells. Occasional follicles were observed as intermediate between primordial and primary and had both cuboidal and squamous granulose cells. If cuboidal cells predominated follicles were classified as the primary type. Follicles were classed as secondary if they possessed more than one layer of granulosa cells with no visible antrum. Antral follicles possessed one or multiple visible fluid-filled antral cavities. Preovulatory follicles had a rim of cumulus cells surrounding the oocyte. *Corpus luteum* is typically very large relative to the size of the ovary and only theca cells and granulosal cells were observed. Follicles with degeneration of oocytes were judged as atresia.

### 4.5. Estrous Cycle Monitoring

After the treatment, vaginal cell samples were taken each day for 28 days. Briefly speaking, plastic pipette filled with saline (~10 μL) was placed into the vagina. The vagina was gently flushed three to five times with same saline solution. Final flush was collected and placed on a glass slide. Unstained material was observed under a light microscope with a 10× objective. Mice were judged as being in one of four stages on the basis of the cellular profile of each smear: proestrus (80%~100% intact, live epithelial cells), estrus (100% cornified epithelia), metestrus (50% cornified epithelia and 50% leukocytes), or diestrus (80%~100% leukocytes).

### 4.6. Serum Hormone Assay

Blood was collected at a proestrus period by removing the eyeballs. Blood samples were placed in 1.5 ml microcentrifuge tubes at 4 °C overnight. On the second day, the tubes were centrifuged at 1500 rpm for 30 min at 4 °C and the serums were collected. The serums were stored at −80 °C until the hormone assays. Estradiol and progesterone levels were measured in plasma samples using a rodent ELISA kit according to the manufacturer’s instructions (Enzo Life Sciences, Lausen, Switzerland). The limits of sensitivity of the assays were 14.0 pg/mL for estradiol and 0.1 ng/mL for progesterone.

### 4.7. Assessment of Fertility

MC-LR was administrated for three or six months before mating. Non-treated male mice were used only for mating purposes. Female mice were cohabited with non-treated male every night and successful mating was judged by detecting a vaginal plug. The daily dose of MC-LR was continued during the daytime. The number of litters and pups were recorded over a three months period.

### 4.8. Mouse Primary Granulosa Cell Collection and Culture

Mouse granulosa cells (mGCs) were collected from the ovaries of 21-day-old immature BALB/c mice using the follicle puncture method, as described by Kipp *et al*. [46]. Oocytes were filtered out with a 40-μm cell strainer. The mGCs were cultured in DMEM-F12 supplemented with 10% FBS and antibiotics (100 IU/mL penicillin, 100 mg/mL streptomycin obtained from Sigma). The cells were incubated at 37 °C with 5% CO_2_ in a humidified chamber.

### 4.9. Cell Viability Assay

Cell viability was evaluated by the Cell Counting Kit-8 (CCK-8). CCK-8 contains WST-8 which can be deoxidized to hydrosoluble Formazan dye by mitochondrial dehydrogenase in living cells. Briefly, purified mGCs were seeded into to 96-well culture plates 100 μL per well at a density of 1 × 10^5^ cells/ml. After incubated for 48 h, cells were exposed to different concentrations of MC-LR (0, 0.625, 1.25, 2.5, 5, 10, 20, 40 μM), respectively. Then cells were incubated in a humidified atmosphere of 95% air, 5% CO_2_ at 37 °C. After incubation for 48 h, 10 μL CCK-8 solution was added to each well and incubated at 37 °C for 4 h. The absorbance, expressed as optical density (OD), was measured at 450 nm with a multidetection microplate reader (Versamax, Chester, PA, USA). This experiment was repeated three times in sextuplicate.

### 4.10. Immunofluorescence Detection

Purified mGCs were seeded into a six-well culture plate and maintained in a humidified atmosphere of 95% air, 5% CO_2_ at 37 °C. After 48 h incubation, the cells were treated with MC-LR at the concentration of 1.25 μM and incubated for 48 h. Then mGCs were collected, centrifuged at 1200 rpm for 5 min at 4 °C and incubated sequentially with 4% paraformaldehyde fixative for 10 min and 3% BSA blocking agents for 30 min. Primary antibody (monoclonal antibody to MC-LR diluted by 3% BSA with 1:200 dilution) was then applied and incubated overnight. The fluorochrome conjugated secondary antibody (Alexa Fluor^®^ 594 donkey anti-mouse IgG (H+L) with 1:200 dilution) was added and samples were incubated for 1 h at 34 °C. Nuclei were counterstained with 5 μg/mL DAPI. Finally, the slides were mounted with glycerin and observed using a confocal microscopy (Fluoview FV10i, Tokyo, Japan) with a 600× objective.

### 4.11. Determination of Lipid Peroxidation and Antioxidant Enzymes

Purified mGCs (1 × 10^5^/mL) were seeded into 24-well plates. 48 h later, mGCs were exposed to MC-LR at 0, 1.25, 5 and 20 μM, respectively. After 48 h exposure, mGCs were harvested, washed twice with PBS, and lysed in cell lysis buffer, centrifuged at 12,000× *g* for 10 min at 4 °C, and then the supernatant was collected. Lipid peroxidation levels were determined by measuring MDA content spectrophotometrically at 532 nm (results expressed as nmol/mg protein), an end-product of lipid peroxidation [47]. CAT activity was evaluated by measuring the decomposition of hydrogen peroxide spectrophotometrically at 520 nm [48]. SOD activity was determined by measuring the inhibition of pyrogallol auto-oxidation spectrophotometrically at 450 nm [49]. CAT and SOD activity was expressed as U/mg protein. All the operation process according to the manufacturer's instructions and measured with a Microplate Reader. The protein concentration of each treatment group was determined using the BCA protein assay kit (Beyotime).

### 4.12. Statistical Analysis

The data are expressed as the mean ± standard error (S.E.). All statistical analyses were carried out via SPSS for windows version 17. One-way analysis of variance (ANOVA) was used to analyze the difference between groups, followed by Dunnett’s *t*-test. *p* < 0.05 was regarded as statistically significant.

## 5. Conclusions

Our data indicates that chronic exposure to MC-LR exhibited strong female reproductive toxicity and induce subfertility in female mice. As the crucial supporting somatic cells of follicle, mGCs are the target of MC-LR through uptaking the toxicant. Oxidative stress in mGCs induced by MC-LR promoted follicle atresia and eventually leads to female subfertility.

## Supplementary Materials

The following are available online at www.mdpi.com/2072-6651/7/12/4872/s1.
