# Supplementary material for: MC-LR Exposure Leads to Subfertility of Female Mice and Induces Oxidative Stress in Granulosa Cells
Source: Toxins (Basel). 2015 Dec 2;7(12):5212–23. doi: 10.3390/toxins7124872 (PMC4690122; doi:10.3390/toxins7124872)
Supplement: Supplementary file 1 [file toxins-07-04872-s001.pdf]

Supplementary Materials: MC-LR Exposure Leads to Subfertility of Female Mice and Induces Oxidative Stress in Granulosa Cells

Jiang Wu, Mingming Yuan, Yuefeng Song, Feng Sun and Xiaodong Han

Experimental Section

HPLC-MS/MS Measurement of MC-LR

The HPLC system consisted of one LC-20AB ultra-high pressure gradient pump, a vacuum degasser (DGU-20A5), and an autosampler (SIL-20AC). Column temperature was maintained at 20 °C. The samples were injected onto a 3 × 100-mm Inert Sustain C18 column, 3 μm diameter. The MC-LR was eluted in solution (0.3% HAc: 100% Acetonitrile = 50:50). The flow rate was set at 0.2 mL/min. For each analysis, 1 μL of sample was injected onto the column, and the total analysis time, including the equilibration, was 6.5 min. A LCMS-8040 (Shimadzu Corporation, Kyoto, Japan) was used in the positive electrospray ionization mode. Nitrogen was used as the nebulizing gas, and argon was used as the collision gas. The MC-LR was detected with the mass spectrometer in Multiple Reaction Monitoring (MRM) mode. The MRM transition for MC-LR was as follows: MC-LR (parent ion *m*/*z*: 955.7 to product ion *m*/*z*: 135.2) (Figure S1).

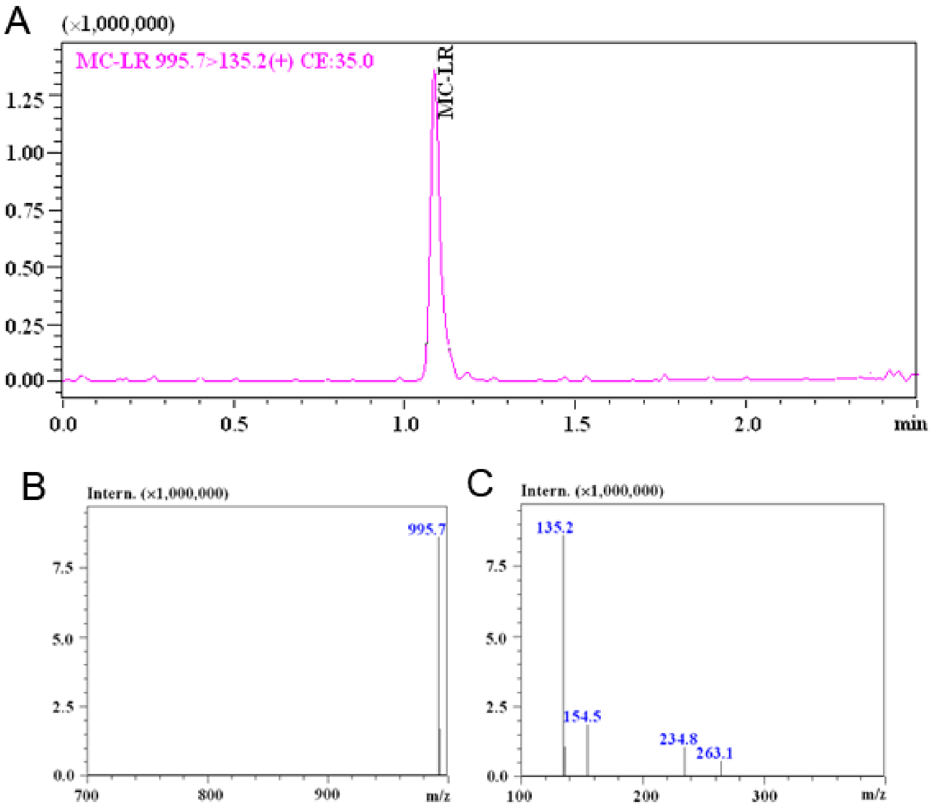

Figure S1. LC–MS/MS detection for MC-LR. (**A**) MC-LR MRM chromatogram; (**B**) MC-LR parent ion mass spectrum; (**C**) MC-LR product ion mass spectrum.
